# Evolution of Scaling Emergence in Large-Scale Spatial Epidemic Spreading

**DOI:** 10.1371/journal.pone.0021197

**Published:** 2011-07-01

**Authors:** Lin Wang, Xiang Li, Yi-Qing Zhang, Yan Zhang, Kan Zhang

**Affiliations:** Adaptive Networks and Control Lab, Department of Electronic Engineering, Fudan University, Shanghai, People's Republic of China; Universidad Veracruzana, Mexico

## Abstract

**Background:**

Zipf's law and Heaps' law are two representatives of the scaling concepts, which play a significant role in the study of complexity science. The coexistence of the Zipf's law and the Heaps' law motivates different understandings on the dependence between these two scalings, which has still hardly been clarified.

**Methodology/Principal Findings:**

In this article, we observe an evolution process of the scalings: the Zipf's law and the Heaps' law are naturally shaped to coexist at the initial time, while the crossover comes with the emergence of their inconsistency at the larger time before reaching a stable state, where the Heaps' law still exists with the disappearance of strict Zipf's law. Such findings are illustrated with a scenario of large-scale spatial epidemic spreading, and the empirical results of pandemic disease support a universal analysis of the relation between the two laws regardless of the biological details of disease. Employing the United States domestic air transportation and demographic data to construct a metapopulation model for simulating the pandemic spread at the U.S. country level, we uncover that the broad heterogeneity of the infrastructure plays a key role in the evolution of scaling emergence.

**Conclusions/Significance:**

The analyses of large-scale spatial epidemic spreading help understand the temporal evolution of scalings, indicating the coexistence of the Zipf's law and the Heaps' law depends on the collective dynamics of epidemic processes, and the heterogeneity of epidemic spread indicates the significance of performing targeted containment strategies at the early time of a pandemic disease.

## Introduction

Scaling concepts play a significant role in the field of complexity science, where a considerable amount of efforts is devoted to understand these universal properties underlying multifarious systems[1]–[4]. Two representatives of scaling emergence are the Zipf's law and the Heaps' law. G.K. Zipf, sixty years ago, found a power law distribution for the occurrence frequencies of words within different written texts, when they were plotted in a descending order against their rank[5]. This frequency-rank relation also corresponds to a power law probability distribution of the word frequencies[32]. The Zipf's law is found to hold empirically for a great deal of complex systems, e.g., natural and artificial languages[5]–[9], city sizes[10], [11], firm sizes[12], stock market index[13], [14], gene expression[15], [16], chess opening[17], arts[18], paper citations[19], family names[20], and personal donations[21]. Many mechanisms are proposed to trace the origin of the Zipf's law[22]–[24].

Heaps' law is another important empirical principle describing the sublinear growth of the number of unique elements, when the system size keeps on enlarging[25]. Recently, particular attention is paid to the coexistence of the Zipf's law and the Heaps' law, which is reported for the corpus of web texts[26], keywords in scientific publication[27], collaborative tagging in web applications[28], [29], chemoinformatics[30], and more close to the interest in this article, global pandemic spread[31], and etc.

In [33], [34], an improved version of the classical Simon model[35] was put forward to investigate the emergence of the Zipf's law, which is deemed to be a result from the existence of the Heaps' law. However, [26], [32] concluded that the Zipf's law leads to the Heaps' law. In fact, the interdependence of these two laws has hardly been clarified. This embarrassment comes from the fact that the empirical/simulated evidence employed to show the emergence of Zipf's law mainly deals with static and finalized speicmens/results, while the Heaps' law actually describes the evolving characteristics.

In this article, we investigate the relation between these scaling laws from the perspective of coevolution between the scaling properties and the epidemic spread. We take the scenarios of large-scale spatial epidemic spreading for example, since the empirical data contain sufficient spatiotemporal information making it possible to visualize the evolution of the scalings, which allows us to analyze the inherent mechanisms of their formation. The Zipf's law and the Heaps' law of the laboratory confirmed cases are naturally shaped to coexist during the early epidemic spread at both the global and the U.S. levels, while the crossover comes with the emergence of their inconsistency as the epidemic keeps on prevailing, where the Heaps' law still exists with the disappearance of strict Zipf's law. With the U.S. domestic air transportation and demographic data, we construct a fine-grained metapopulation model to explore the relation between the two scalings, and recognize that the broad heterogeneity of the infrastructure plays a key role in their temporal evolution, regardless of the biological details of diseases.

## Results

### Empirical and Analytical Results

**Figure 1:**
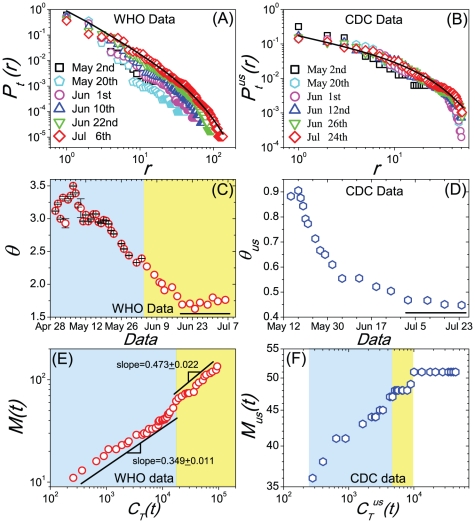
The empirical results of A(H1N1). (A) The Zipf-plots of the normalized probability-rank distributions 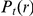 of the cumulated confirmed number of every infected country at several given date sampled about every two weeks, data provided by the WHO. (B) The Zipf-plots of 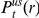 at several given data sampled about every two weeks, data provided by the CDC. (C) Temporal evolution of the estimated exponent 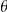 of the normalized distribution 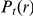. (D) Temporal evolution of the estimated exponent 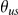 of the normalized distribution 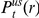 of the period after May 15th. (E) The sublinear relation between the number of infected countries 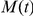 and the cumulative number of global confirmed cases 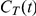, data collected by the WHO. (F) The sublinear relation between the number of infected states 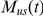 and the cumulative number of national confirmed cases 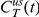, data collected by the CDC. The shaded areas in the figures (C,E,F) corresponds to their different evolution stages, respectively.

With the empirical data of the laboratory confirmed cases of the A(H1N1) provided by the World Health Organization(WHO)(see the data description in *Materials and Methods*), we first study the probability-rank distribution(*PRD*) of the cumulative confirmed number(*CCN*) of every infected country at several given dates sampled about every two weeks. 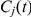 denotes the *CCN* in a given country 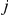 at time 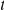. Since 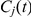 grows with time, the distributions at different dates are normalized by the global *CCN*, 
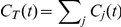
, for comparison. Fig. 1(A) shows the Zipf-plots of the *PRD* 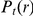 of the infected countries' confirmed cases by arranging every 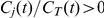 in a descending order for each specimen. The maximal rank 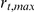
(on x-axis) for each specimen denotes the total number of infected countries at a given date, and grows as the epidemic spreading.

At the early stage(the period between April 30th and June 1st, 2009), 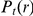 shows a power law pattern 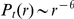, which indicates the emergence of the Zipf's law. We estimate the power law exponent 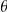 for each specimen of this stage by the maximum likelihood method[22], [37], and report its temporal evolution in the left part of Fig. 1(C). About sixty countries were affected by the A(H1N1) on June 1st, and most of them are countries with large population and/or economic power, e.g., U.S., Mexico, Canada, Japan, Australia, China. After June 1st, the disease swept much more countries in a short time, and the WHO announcement on June 11th[38] raised the pandemic level to its highest phase, phase 6(see *Text S1*), which implied that the global pandemic flu was occurring. At this stage(after June 1st, 2009), 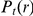 gradually displays a power law distribution with an exponential cutoff 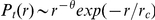, where 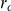 is the parameter controlling the cutoff effect(see *Text S1*), and the exponent 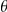 gradually reduces to around 1.7, as shown in Fig. 1(C). Surprisingly, 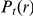 at different dates eventually reaches a stable distribution as time evolves(see those curves since June in Fig. 1(A)). Indeed, after June 19th, 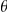 seems to reach a stable value with mild fluctuations, as shown in Fig. 1(C). The characteristics of the temporal evolution of the parameter 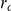 is similar to 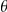, thus we mainly present the empirical results of the exponent 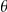 in the main text and hold the results of 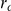 in Figure S1. In the following, we analyze the evolution of the normalized distribution 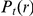 by the contact process of an epidemic transmission, regardless of the biological details of diseases.

Straightforwardly, according to the mass action principle in the mathematical epidemiology[39], [40](see *Text S1*), which is widely applied in studying the epidemic spreading process on a network[41]–[56], we consider the SIR epidemic scheme here,
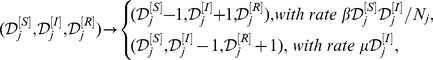
(1)


where 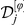 denotes the number of individuals in compartment 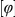
(susceptible(S), infectious(I) or permanently recovered(R)) in a given country 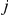, 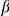 denotes the disease transmission rate, and infectious individuals recover with a probability 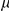. The population in a given country 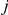 at time 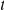 is 
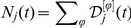
, where 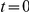 means the time when initially confirmed cases in the entire system are reported. At the early stage of a pandemic outbreak, the new introductions of infectious individuals dominate the onset of outbreak in unaffected countries. However, after the disease already lands in these countries, the ongoing indigenous transmission gradually exceeds the influence of the new introductions, and becomes the mainstream of disseminators[57], [58]. According to Eq.(1), in a given infected country 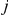, there are
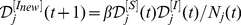
(2)


new infected individuals on average at 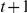 days, and the average number of illness at 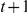 days is
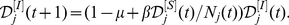
(3)


Defining 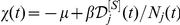 and 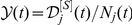, we have
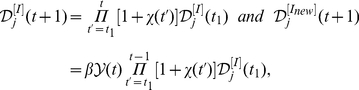
(4)


where 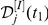 denotes the number of initially confirmed or introduced cases in country 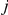, and is always a small positive integer. The *CCN* of country 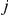 at 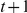 days is 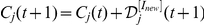. When 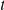 is large enough, we have
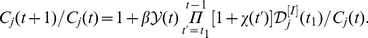
(5)


Before the disease dies out in country 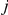, 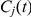 keeps increasing from the onset of outbreak[59]. When 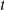 is large enough, it is obviously 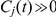, 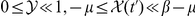, thus 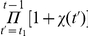 is definitely larger than 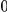 and can hardly be infinity. 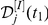 is a small positive integer, thus 
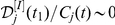
 when 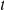 is large enough. We therefore have 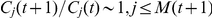 for large 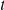, where 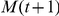 is the total number of infected countries after 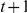 days of spreading. Thus the normalized probability 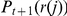 at 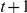 day is:
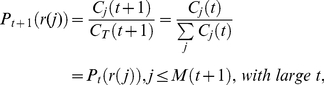
(6)


where 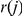 is the rank of the *CCN* of country 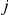 in the descending order of the *CCN* list of all infected countries. Eq.(6) indicates that each probability 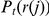 is invariant for large 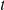, thus the normalized distribution 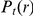 becomes stable when 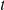 is large enough. The intrinsic reasons for the emergence of these scaling properties are discussed in *Modeling and Simulation Results*.

Since the normalized *PRD* 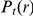 displays the Zipf's law pattern 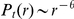 at the early stage of the epidemic, the *CCN* of the country ranked 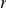 is 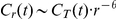 at this stage. Considering the *CCN* of the countries with ranks between 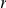 and 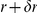, where 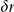 is any infinitesimal value, we have 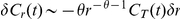. Supposing 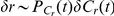 with 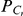 denoting the probability density function, we have
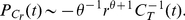
(7)


Thus
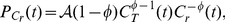
(8)


where 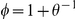, 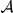 is a constant. According to the normalization condition 
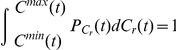
, where 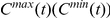 is the *CCN* of the country with the maximal(minimal) value at a give time 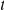, we have 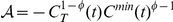 because 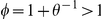 and 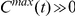. Then
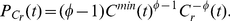
(9)


At a given date, 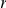 can be regarded as the number of countries with the amount of cumulated confirmed cases which is no less than 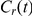, then
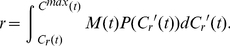
(10)


Recalling 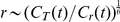, we have
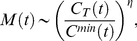
(11)


where 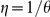. At the early stage corresponding to the period between April 30th and June 1st, 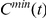 is one according to the WHO data. Therefore, we have
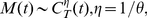
(12)


which indicates that the Heap's law[25], [26], [31], [32] can be observed in this case. The empirical evidence for the emergence of the Heap's law at this stage is shown in the middle part of Fig. 1(E). The Heaps' exponent 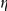 is obtained by the least square method[31], [32], and the relevance between 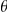 and 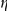 is reported in Table 1.

**Table 1:** The empirical results of the parameters 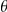 and 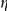, and their relevance at the early time(the period between April 30th and June 1st, 2009), using 2009 Pandemic A(H1N1) data collected by the WHO.

Date	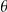	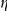	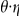
April 30th	3.12	0.349	1.046
May 1st	3.23	0.349	1.127
May 2th	3.00	0.349	1.047
May 3th	3.32	0.349	1.159
May 4th	2.93	0.349	1.022
May 5th	3.29	0.349	1.148
May 6th	3.35	0.349	1.169
May 7th	3.5	0.349	1.222
May 8th	3.39	0.349	1.183
May 9th	3.2	0.349	1.117
May 10th	3.16	0.349	1.103
May 11th	2.96	0.349	1.033
May 12th	3.06	0.349	1.068
May 13th	2.96	0.349	1.033
May 14th	3.00	0.349	1.047
May 15th	3.07	0.349	1.071
May 16th	3.07	0.349	1.071
May 17th	2.95	0.349	1.030
May 18th	2.93	0.349	1.023
May 19th	2.98	0.349	1.040
May 20th	2.97	0.349	1.037
May 21th	2.92	0.349	1.019
May 22th	2.82	0.349	0.984
May 23th	2.77	0.349	0.967
May 26th	2.62	0.349	0.914
May 27th	2.54	0.349	0.886
May 29th	2.44	0.349	0.852
June 1st	2.33	0.349	0.813

At the latter stage(the period after June 1st, 2009), the exponential tail of the distribution 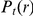 leads to a deviation from the strict Zipf's law. However, with a steeper exponent 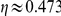, the Heaps' law still exists, as shown in the right part of Fig. 1(E). Though the two scaling laws are naturally shaped to coexist during the early epidemic spreading, their inconsistency gradually emerges as the epidemic keeps on prevailing. Indeed, in the *Discussion* of [32], without empirical or analytical evidence, Lü et al have intuitively suspected that there may exist some unknown mechanisms only producing the Heaps' law, and it is possible that a system displaying the Heaps' law does not obey the strict Zipf's law. Here we not only verify this suspicion with the empirical results, but also explore the substaintial mechanisms of the evolution process in *Modeling and Simulation Results*, where we uncover the important role of the broad heterogeneity of the infrastructure in the temporal evolution of scaling emergence.

We also empirically study the evolution of scaling emergence of the epidemic spreading at the countrywide level. Since the United States is one of the several earliest and most seriously prevailed countries of the A(H1N1)[60], we mainly focus on the A(H1N1) spreading in the United States. With the empirical data of the laboratory confirmed cases of the A(H1N1) provided by the Centers for Disease Control and Prevention(CDC)(see the data description in *Materials and Methods*), in Fig. 1(B) we report the *PRD* of the *CCN* of infected states, 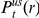, at several given dates sampled about every two weeks. Our findings suggest a crossover in the temporal evolution of 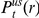. At the early stage(the period before May 15th), 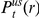 shows a power law pattern 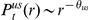 with a much smaller exponent 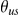 than that of the WHO results. Washington D.C. and 46 states(excluding Alaska, Mississippi, West Virginia, Wyoming) were affected by A(H1N1) on May 15th. After May 15th, 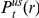 gradually becomes a power law distribution with an exponential cutoff, 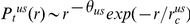, which leads to a deviation from the strict Zipf's law. In this case, the exponent 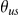 gradually reduces and reaches a stable value 0.45(see Fig. 1(D)), which conforms to the fact that 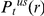 of different dates eventually reaches a stable distribution as time evolves. The temporal evolution of the exponent 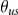 of all data are shown in Figure S2. 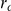 keeps the value around 14 after June 12th, 2009.

The relation between 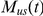 and 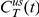 is shown in Fig. 1(F). Though at first glance this figure provides us an impression of the sublinear growth of the number of infected states 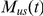 when the cumulative number of national total patients 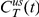 increases, we could not use the least square method here to estimate the Heaps' exponent 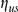 for several reasons: (i) the amount of data at each stage is quite small; (ii) there are several periods that 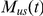 keeps unchanged(May 6th 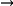 May 7th, 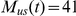; May 12th 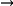 May 13th, 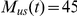; May 18th 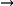 May 27th, 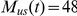); (iii) the magnitude of 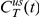 is much larger than that of 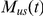; (iv) after June 1st, 2009, Washington D.C. and all 50 states of the United States were affected by the A(H1N1). Define 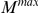 the maximal number of the geographical regions the epidemic spreads to. In the U.S. scenario, 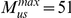. When 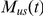 reaches 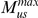 on June 1st, 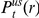 evolves and becomes stable after June 26th(see Fig. 1(B,D)). In the *Modeling and Simulation Results*, we explore the relation between these two scalings with a fine grained metapopulation model characterizing the spread of the A(H1N1) at the U.S. level in detail.

Note that these scaling properties are not exceptive for the A(H1N1) transmission. More supported exemplifications are reported in *Figure S3*, e.g. the cases of SARS, Avian Influenza(H5N1). It is worth remarking that the normalized distribution 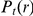 almost keeps the power law pattern during the whole spreading process of the global SARS. This phenomenon might result from the intense containment strategies, e.g. patient isolation, enforced quarantine, school closing, travel restriction, implemented by individuals or governments confronting mortal plague.

### Modeling and Simulation Results

The above analyses, however, do not tell the whole story, because the intrinsic reasons for the emergence of these scaling properties have not been explained. Some additional clues from the perspective of Shannon entropy[61] of a system might unlock the puzzle.

Nowadays, population explosion in the urban areas, massive interconnectivity among different geographical regions, and huge volume of human mobility are the factors accelerating the spread of infectious disease[62], [74]. At a large geographical scale, one main class of models is the metapopulation model dividing the entire system into several interconnected subpopulations[58], [63]–[74], [87], [88]. Within each subpopulation, the infectious dynamics is described by the compartment schemes, while the spread from one subpopulation to another is due to the transportation and mobility infrastructures, e.g., air transportation. Individuals in each subpopulation exist in various discrete health compartments(status), i.e. susceptible, latent, infectious, recovered, and etc., with compartmental transitions by the contagion process or spontaneous transition, and might travel to other subpopulations by vehicles, e.g., airplane, in a short time. The metapopulation model can not only be employed to describe the global pandemic spread when we regard each subpopulation as a given country, but also be used to simulate the disease transmission within a country when each subpopulation is regarded as a given geographical region in the country. Here we mainly consider the spread of pandemic influenza at the U.S. country level for threefold reasons: (i) the computational cost of simulating global pandemic spread is too tremendous to implement on a single PC or Server[58], [70], [72], [81], [87]; (ii) the IATA or OAG flight schedule data, which is widely used to obtain the global air transportation network, do not provide the attendance and flight-connecting information(see data description in *Materials and Methods*); (iii) the United States is one of the several earliest and most seriously prevailed countries[60].

**Figure 2:**
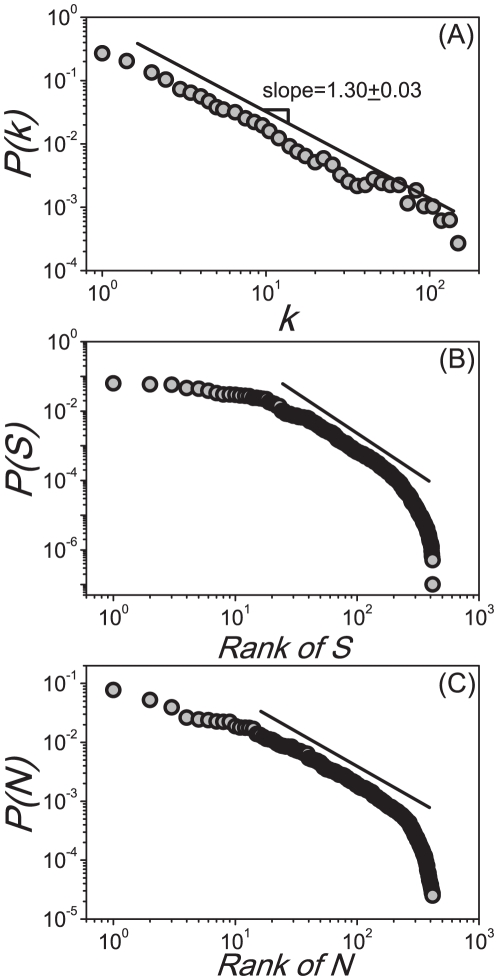
The heterogeneity of the USDAN's infrastructure. (A) The degree distribution 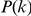 follows a power law pattern on almost two decades with an exponent 1.30
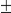
0.03. (B) shows that the probability-rank distribution of the traffic outflux 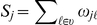, where 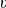 denotes the set of neighbors belonging to the vertex 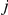 and the weight 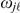 of a connection between two vertices 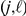 is the number of passengers traveling a given route per day, is skewed and heterogeneously distributed. (C) shows that the probability-rank distribution of populations is skewed and heterogeneously distributed.

We construct a metapopulation model at the U.S. level with the U.S. domestic air transportation and demographic statistical data[75]–[78](detailed data description is provided in *Materials and Methods*, and a full specification of the simulation model is reported in *Text S1*). Define a subpopulation as a Metropolitan/Micropolitan Statistical Areas(MSAs/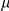
SAs)[75] connected by a transportation network, in this article, the U.S. domestic airline network(USDAN). The USDAN is a weighted graph comprising 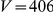 vertices(airports) and 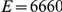 weighted and directed edges denoting flight courses. The weight of each edge is the daily amount of passengers on that flight course. The infrastructure of the USDAN presents high levels of heterogeneity in connectivity patterns, traffic capacities and population(see Fig. 2). The disease dynamics in a single subpopulation is modeled with the Susceptible-Latent-Infectious-Recovered(SLIR) compartmental scheme, where the abbreviation L denotes the latent compartment which experiences 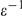 days on average for an infected person(The SIR epidemic dynamics discussed at *Empirical and Analytical Results* is an reasonable approximation, which actually simplifies the epidemic evolution to a Markov chain to help us study the issue, and the value of the reproductive number 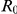 does not depend on 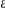, we therefore ignore the compartment L there).

The key parameters determining the spreading rate of infections are the reproductive number 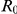 and the generation time 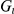. 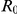 is defined as the average amount of individuals an ill person infects during his or her infectious period 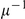 in a large fully susceptible population, and 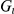 refers to the sum of the latent period 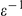 and the infectious period 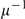. In our metapopulation model, 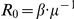. The initial conditions of the disease are defined as the onset of the outbreak in San Diego-Carlsbad-San Marcos, CA MSA on April 17th, 2009, as reported by the CDC[79]. Assuming a short latent period value 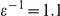 days as indicated by the early estimates of the pandemic A(H1N1)[80], which is compatible with other recent studies[81], [82], we primarily consider a baseline case with parameters: 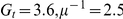 days and 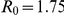, which are higher than those obtained in the early findings of the pandemic A(H1N1)[80], but they are the median results in other subsequent analyses[81], [83]. Fixing the latency period to 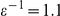 days, we also employ a more aggravated baseline scenario with parameters: 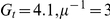 days and 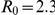, which are close to the upper bound results in[81], [83]–[85].

In succession, we characterize the disease spreading pattern by information entropy, which is customarily applied in information theory. To quantify the heterogeneity of the epidemic spread at the U.S. level, we examine the prevalence at each time 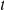, 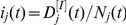, for all subpopulations, and introduce the normalized vector 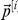 with components 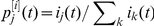. Then we measure the level of heterogeneity of the disease prevalence by quantifying the disorder encoded in 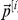 with the normalized entropy function
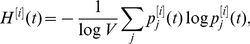
(13)


which provides an estimation of the geographical heterogeneity of the disease spread at time 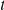. If the disease is uniformly influencing all subpopulations(e.g., all prevalences are equivalent), the entropy reaches its maximum value 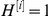. On the other hand, starting from 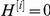, which is the most localized and heterogeneous situation that just one subpopulation is initially affected by the disease, 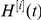 increases as more subpopulations are influenced, thus decreasing the level of heterogeneity.

In order to better uncover the origin of the emergence of the scaling properties, we compare the baseline results with those obtained on a null model *UNI*. The *UNI* model is a homogeneous Erdös-Rényi random network with the same number of vertices as that of the USDAN, and the generating regulation is described as follows: for each pair of vertices 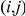, an edge is independently generated with the uniform probability 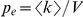, where 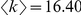 is the average out-degree of the USDAN. Moreover, the weights of the edges and the populations are uniformly equal to their average values in the USDAN, respectively. Therefore, the *UNI* model is completely absent from the heterogeneity of the airline topology, flux and population data.

**Figure 3:**
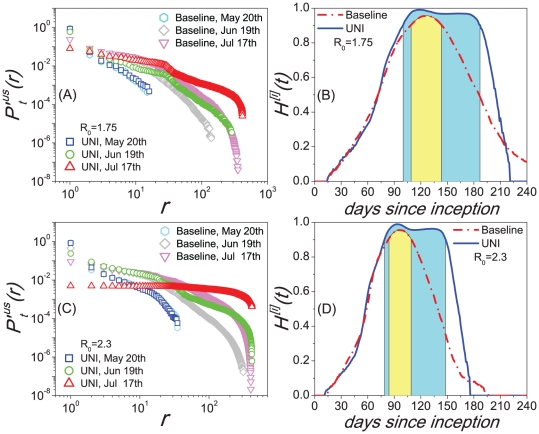
Comparisons of the scaling properties between the *UNI* scenarios and the baseline cases. (A,C) present the comparison of the *PRD* 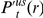 of the *CCN* of every infected *MSA/* 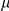 *SA* between the baselines and the *UNI* scenarios at several given date sampled about every 30 days when 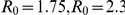, respectively. (B,D) present the comparison of the information entropy profiles between the baselines and the *UNI* results when 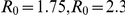, respectively. Each data in these figures are the median results over all runs that led to an outbreak at the U.S. level in 100 random Monte Carlo realizations.

Different evolving behaviors between the *UNI* scenarios and the baselines(real airline cases) provide a remarkable evidence for the direct dependence between the scaling toproperties and the heterogeneous infrastructure. Fig. 3(A,C) show the comparison of the *PRD* between the baseline results and the *UNI* outputs at several given dates sampled about every 30 days, where each specimen is the median result over all runs that led to an outbreak at the U.S. level in 100 random Monte Carlo realizations. In Fig. 3(A), we consider the situation of 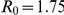, and do observe that the evolution of *PRD* of the baseline case experiences two stages: a power law at the initial time and an exponentially cutoff power law at a larger time. However, the *UNI* scenario shows a distinct pattern: as time evolves, the middle part of the *PRD* grows more quickly, and displays a peak which obviously deviates scaling properties. Fig. 3(C) reports the situation of 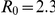. In this aggravated instance, the *PRD* of the *UNI* scenario actually becomes rather homogeneous when 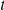 is large enough(see the curve of July 17th of the *UNI* scenario in Fig. 3(C)). Fig. 3(B,D) present the comparison of the information entropy profiles between the baseline results and the *UNI* outputs when 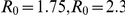, respectively. The completely homogeneous network *UNI* shows a homogeneous evolution(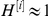) of the epidemic spread in a long period(see the light cyan areas in Fig. 3(B,D)), with sharp fallings at both the beginning and the end of the outbreak. However, we observe distinct results in the baselines, where 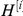 is significantly smaller than 1 for most of the time, and the long tails indicate a long lasting heterogeneity of the epidemic prevalence. These analyses signal that the broad heterogeneity of infrastructure plays an essential role in the emergence of scalings.

**Figure 4:**
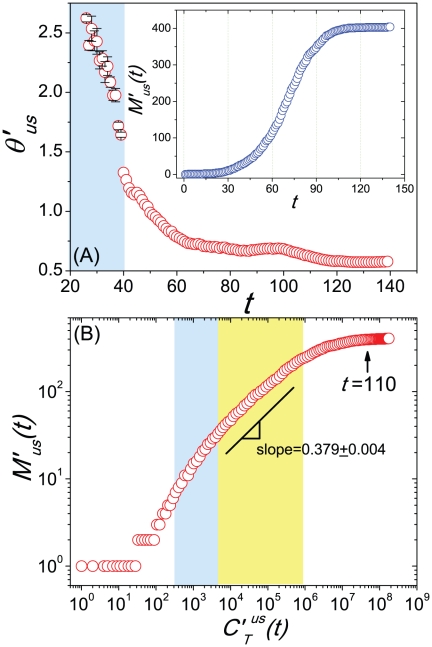
The statistical results of the scaling properties of our metapopulation model. (A) Temporal evolution of the estimated exponent 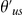 of the normalized distribution 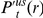. The inset shows the growing of the number of infected subpopulations 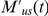 with time 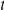. (B) The relation between the number of infected subpopulations 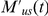 and the national cumulative confirmed cases 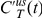. The shaped areas in the figures corresponds to their different evolution stages, respectively. Each data in these figures are the median results over all runs that led to an outbreak at the U.S. level in 100 random Monte Carlo realizations.

We further explore the properties of the two scalings and their relation with the baseline case of 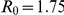 in detail. Since each independent simulation generates a stochastic realization of the spreading process, we analyze the statistical properties with 100 random Monte Carlo realizations, measure the normalized *PRD* of the *CCN* of infected MSAs/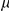
SAs for each realization that led to an outbreak at the U.S. level, and report the median result of the *PRD* 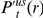 of each day. From 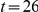 to 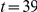, 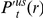 clearly shows a power law pattern 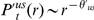, which implies the emergence of the Zipf's law(when 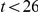, just several regions are affected by the disease). The exponent 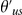 at each date is estimated by the maximum likelihood method[22], [37], and the temporal evolution of 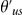 is reported in the left part of Fig. 4(A). When 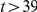, 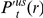 gradually becomes an exponentially cutoff power law distribution 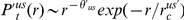, and the exponent 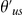 gradually reduces and reaches a stable value of 0.574 with neglectable fluctuations when 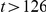
(see Fig. 4(A)). Here we do not show the error bar since the fitting error on the exponent is far less(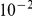) than the value of 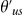 by the average of 100 random realizations. The inset of Fig. 4(A) shows the increase of the number of infected regions 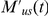 as time evolves. When 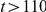, more than 400 subpopulations reports the existence of confirmed cases, thus 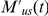 tends to reach its saturation.

Fig. 4(B) shows the relation between 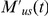 and 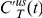
(the national cumulative number of patients). Since 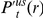 displays a power law of 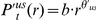 at the early stage of the period between 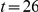 and 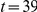, it is reasonable to deduce the existence of the Heaps' law
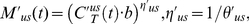
(14)


according to the analyses in *Empirical and Analytical Results*. In order to verify this assumption, we estimate the exponent 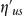 using Eq.(14), and report the relevance between 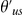 and 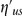 in Table 2(the amount of data in this period is not sufficient to get a accurate estimation of the exponent 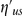 with the least square method). When 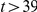, though 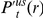 gradually deviates the strict Zipf's law, the Heaps' law of the relation between 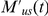 and 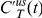 still exists till 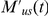 tends to reach its saturation(see the middle part in Fig. 4(B)).

**Table 2:** The value of the parameters 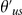 and 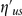 for the simulation results at the early time of the period between 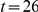 and 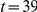.

*t*	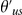	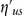	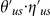
26	2.623	0.427	1.120
27	2.395	0.459	1.099
28	2.535	0.449	1.138
29	2.433	0.457	1.112
30	2.429	0.456	1.108
31	2.269	0.455	1.032
32	2.285	0.460	1.051
33	2.170	0.482	1.046
34	2.220	0.477	1.059
35	2.086	0.492	1.026
36	1.976	0.503	0.994
37	1.977	0.504	0.996
38	1.717	0.540	0.927
39	1.644	0.538	0.884

## Discussion

Zipf's law and Heaps' law are two representatives of the scaling concepts in the study of complexity science. Recently, increasing evidence of the coexistence of the Zipf's law and the Heaps' law motivates different understandings on the dependence between these two scalings, which is still hardly been clarified. This embarrassment derives from the contradiction that the empirical or simulated materials employed to show the emergence of Zipf's law are often finalized and static specimens, while the Heaps' law actually describes the evolving characteristics.

In this article, we have identified the relation between the Zipf's law and the Heaps' law from the perspective of coevolution between the scalings and large-scale spatial epidemic spreading. We illustrate the temporal evolution of the scalings: the Zipf's law and the Heaps' law are naturally shaped to coexist at the early stage of the epidemic at both the global and the U.S. levels, while the crossover comes with the emergence of their inconsistency at a larger time before reaching a stable state, where the Heaps' law still exists with the disappearance of strict Zipf's law.

With the U.S. domestic air transportation and demographic data, we construct a metapopulation model at the U.S. level. The simulation results predict main empirical findings. Employing information entropy characterizing the epidemic spreading pattern, we recognize that the broad heterogeneity of the infrastructure plays an essential role in the evolution of scaling emergence. These findings are quite different from the previous conclusions in the literature. For example, studying a phenomenologically self-adaptive complete network, Han et al. claimed that scaling properties are dependent on the intensity of containment strategies implemented to restrict the interregional travel[31]. In [36], Picoli Junior et al. considered a simple stochastic model based on the multiplicative process[23], and suggested that seasonality and weather conditions, i.e., temperature and relative humidity, also dominates the temporal evolution of scalings because they affect the dynamics of influenza transmission. In this work, without the help of any specific additional factor, we directly show that the evolution of scaling emergence is mainly determined by the contact process underlying disease transmission on an infrastructure with huge volume and heterogeneous structure of population flows among different geographic regions. (The effects of the travel-related containment strategies implemented in real world can be neglected, since the number of scheduled domestic and international passengers of the U.S. air transportation only declined in 2009 by 5.3% from 2008[86]. In fact, the travel restrictions would not be able to significantly slow down the epidemic spread unless more than 90% of the flight volume is reduced[58], [66], [69], [70], [88].)

In summary, our study suggests that the analysis of large-scale spatial epidemic spread as a promising new perspective to understand the temporal evolution of the scalings. The unprecedented amount of information encoded in the empirical data of pandemic spreading provides us a rich environment to unveil the intrinsic mechanisms of scaling emergence. The heterogeneity of epidemic spread uncovered by the metapopulation model indicates the significance of performing targeted containment strategies, e.g. vaccination of prior groups, targeted antiviral prophylaxis, at the early time of a pandemic disease.

## Materials and Methods

### Data Description

In this article, in order to construct the U.S. domestic air transportation network, we mainly utilize the “*Air Carrier Traffic and Capacity Data by On-Flight Market report(December 2009)”* provided by the Bureau of Transportation Statistics(BTS) database[76]. This report contains 12 months' data covering more than 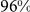 of the entire U.S. domestic air traffic in 2009, and provides the monthly number of passengers, freight and/or mail transported between any two airports located within the U.S. boundaries and territories, regardless of the number of stops between them. This *BTS* report provides a more accurate solution for studying aviation flows between any two U.S. airports than other data sources(the attendance and the flight-connecting information in the OAG flight schedule data are commonly unknown, while the datasets adopted in [63], [64], [66], [69] primarily consider the international passengers). In order to study the epidemic spread in the Continental United States where we have a good probability to select citizens living and moving in the mainland, we get rid of the airports as well as the corresponding flight courses located in Hawaii, and all offshore U.S. territories and possessions from the *BTS* report.

In order to obtain the U.S. demographic data, we resort to the “*OMB Bulletin N0. 10*–*02: Update of Statistical Area Definitions and Guidance on Their Uses”* [75] provided by the United States Office of Management and Budget(OMB), and the “*Annual Estimates of the Population of Metropolitan and Micropolitan Statistical Areas: April 1, 2000 to July 1, 2009”* [77] provided by the United States Census Bureau(CB). OMB defines a Metropolitan Statistical Area(MSA)(Micropolitan Statistical Area, 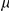
SA) as one or more adjacent counties or county equivalents that have at least one urban core area of at least 50,000 population(10,000 population but less than 50,000), plus adjacent territory that has a high degree of social and economic integration with the core. For other regions with at least 5,000 population but less than 10,000, we use the American FactFinder[78] provided by the CB to get the demographic information. We do not consider sparsely populated areas with population less than 5,000, because they are commonly remote islands, e.g. Block Island in Rhode Island, Sand Point in Alaska.

Before constructing the metapopulation model, we take into account the fact that there might be more than one airport in some huge metropolitan areas. For instance, New York-Northern New Jersey-Long Island(NY-NJ-PA MSA) has up to six airports(their IATA codes: JFK, LGA, ISP, EWR, HPN, FRG), Los Angeles-Long Beach-Santa Ana(CA MSA) has four airports(their IATA codes: LAX, LGB, SNA, BUR), and Chicago-Joliet-Naperville(IL-IN-WI MSA) has two airports(their IATA codes: MDW, ORD). Assuming a homogeneous mixing inside each subpopulation, we need to assemble each group of airports serving the same MSA/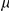
SA, because the mixing within each given census areas is quite high and cannot be characterized by fine-grained version of subpopulations for every single airport. We searched for groups of airports located close to each other and belonged to the same metropolitan areas, and then manually aggregated the airports of the same group in a single “super-hub”.

The full list of updates of the pandemic A(H1N1) human cases of different countries is available on the website of Global Alert and Response(GAR) of World Health Organization(WHO)(WHO website. http://www.who.int/csr/disease/swineflu/updates/en/index.html. Accessed 2011 May 24). It is worth remarking that WHO was no longer updating the number of the cumulated confirmed cases for each country after July 6th, 2009, but changed to report the number of confirmed cases on the WHO Region level(the Member States of the World Health Organization(WHO) are grouped into six regions, including WHO African Region(46 countries), WHO European Region(53 countries), WHO Eastern Mediterranean Region(21 countries), WHO Region of the Americas(35 countries), WHO South-East Asia Region (11 countries), WHO Western Pacific Region(27 countries). (WHO website. http://www.who.int/about/regions/en/index.html. Accessed 2011 May 24).

The cumulative number of the laboratory confirmed human cases of A(H1N1) flu infection of each U.S. state is available at the website of 2009 A(H1N1) Flu of the Centers for Disease Control and Prevention(CDC)(CDC website. http://cdc.gov/h1n1flu/updates/. Accessed 2011 May 24), where the detailed data were started from April 23, 2009, to July 24, 2009. After July 24, the CDC discontinued the reporting of individual confirmed cases of A(H1N1), and began to report the total number of hospitalizations and deaths weekly.

The data of the human cases of global SARS and global Avian influenza(H5N1) are available at the website of the Disease covered by GAR of WHO(WHO website. http://www.who.int/csr/disease/en/. Accessed 2011 May 24).

## Supporting Information

Text S1 (PDF)

Figure S1
**The temporal evolution of the estimated parameter** 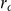 **, data provided by the WHO.**
(EPS)

Figure S2
**The temporal evolution of the estimated exponent** 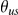 **for all data provided by the CDC.**
(EPS)

Figure S3 **The empirical results of the SARS and avian influenza(H5N1).** (A) shows the normalized probability-rank distribution of the cumulated confirmed number of every infected country around the world at several given date sampled about every four weeks, data provided by the WHO(WHO website. http://www.who.int/csr/sars/country/en/index.html. Accessed 2011 May 24.). (B) shows the normalized probability-rank distribution of the cumulated confirmed number of every infected country around the world at several given date sampled about every half a year, data provided by the WHO(WHO website. http://www.who.int/csr/disease/avian_influenza/country/en/. Accessed 2011 May 24.).
(EPS)
