# Supplementary material for: Evolution of Scaling Emergence in Large-Scale Spatial Epidemic Spreading
Source: PLoS One. 2011 Jul 1;6(7):e21197. doi: 10.1371/journal.pone.0021197 (PMC3128583; doi:10.1371/journal.pone.0021197)
Supplement: Text S1 — (PDF) [file pone.0021197.s001.pdf]

Supporting Information Text S1

"Evolution of scaling emergence in large-scale spatial epidemic spreading"

Lin Wang, Xiang Li∗, Yiqing Zhang, Yan Zhang, Kan Zhang
∗ Corresponding author: lix@fudan.edu.cn

Simulation Model Design

As a basic modeling scheme, we use the metapopulation approach, which explicitly considers the geographical structure in the model by introducing multiple subpopulations coupled by individuals' mobility. More specifically, the subpopulations correspond to the metropolitan areas, and the dynamics of individuals' mobility is described by enplaning between any two regions.

(i) Infection Dynamics in a Single Subpopulation.

The infection dynamics takes place within each single subpopulation, and is described by a homogeneously mixed population with an influenza-like illness compartmentalization in which each individual exists in just one of the following discrete classes such as susceptible(S), latent(L), infectious(I) or permanently recovered(R). In each subpopulation j, the population is N_j, and $\mathcal{D}_j^{[\varphi]}(t)$ is the number of individuals in the class $[\varphi]$ at time t. By definition, it is evident that $N_j = \sum_{\varphi} \mathcal{D}_j^{[\varphi]}(t)$. Two essential kinds of the disease evolution processes are considered in the infection dynamics: the contagion process(e.g., a susceptible individual acquires the infection from any given infectious individual and becomes latent with the rate β, where β is the transmission rate of a disease) and the spontaneous transition of individual from one compartment to another(i.e. latent ones become infectious with a probability ϵ, or the infectious individuals recover with a probability μ, where ϵ^{-1} and μ^{-1} are the average latency time and the average infection duration, respectively). Schematically, the stochastic infection dynamics is given by

$$(\mathcal{D}_j^{[S]}, \mathcal{D}_j^{[L]}, \mathcal{D}_j^{[I]}, \mathcal{D}_j^{[R]}) \Rightarrow \begin{cases} (\mathcal{D}_j^{[S]} - 1, \mathcal{D}_j^{[L]} + 1, \mathcal{D}_j^{[I]}, \mathcal{D}_j^{[R]}), \; with\ rate\ \beta \mathcal{D}_j^{[S]} \mathcal{D}_j^{[I]} / N_j, \\ (\mathcal{D}_j^{[S]}, \mathcal{D}_j^{[L]} - 1, \mathcal{D}_j^{[I]} + 1, \mathcal{D}_j^{[R]}), \; with\ rate\ \epsilon \mathcal{D}_j^{[L]}, \\ (\mathcal{D}_j^{[S]}, \mathcal{D}_j^{[L]}, \mathcal{D}_j^{[I]} - 1, \mathcal{D}_j^{[R]} + 1), \; with\ rate\ \mu \mathcal{D}_j^{[I]}, \end{cases}$$

where the first reaction reflects the fact that each susceptible in subpopulation j would be infected by contacting any infectious individuals with probability $\beta \mathcal{D}_j^{[I]} / N_j$, therefore the number of new infections generated in subpopulation j at time $t + 1$ is extracted from a binomial distribution with the probability $\beta \mathcal{D}_j^{[I]}(t)/N_j(t)$ and the number of trials $\mathcal{D}_j^{[S]}(t)$; the second and the third reactions represent the spontaneous transition process.

(ii)Epidemic Transmission among Different Subpopulations.

As individuals travel around the country, the disease may spread from one area to another. Therefore, in addition to the infection dynamics taking place inside each subpopulation, the epidemic spreading at a large geographical scale is inevitably governed by the human mobility among different subpopulations by means of the domestic air transportation. Since the BTS report reflects the actual aviation flows between any two U.S. airports, we define a stochastic dispersal operator $\nabla_j(\{\mathcal{D}^{[\varphi]}\})$ representing the net balance of individuals in a given compartment $\mathcal{D}^{[\varphi]}$ that entered in and left from each subpopulation j. In each subpopulation j, the dispersal operator is expressed as

$$\nabla_j(\{\mathcal{D}^{[\varphi]}\}) = \sum_{\ell} (\mathcal{X}_{\ell j}(\mathcal{D}_\ell^{[\varphi]}) - \mathcal{X}_{j \ell}(\mathcal{D}_j^{[\varphi]})),$$

where $\mathcal{X}_{j\ell}(\mathcal{D}_j^{[\varphi]})$ describes the daily number of individuals in the compartment $[\varphi]$ traveling from subpopulation j to subpopulation ℓ. In the scenario of air travel, this operator is relative to the passenger traffic flows and the population. Neglecting multiple legs travels and assuming the well mixing of population inside each subpopulation, we deduce that the probability of any individual traveling on each connection $j \to \ell$ everyday is given by $p_{j\ell} = \omega_{j\ell}/N_j$, where $\omega_{j\ell}$ represents the daily passenger number from j to ℓ. The stochastic variables $\mathcal{X}_{j\ell}(\mathcal{D}_j^{[\varphi]})$ therefore follow the multinomial distribution

$$P(\{\mathcal{X}_{j\ell}\}) \quad = \quad \frac{\mathcal{D}_j^{[\varphi]}!}{(\mathcal{D}_j^{[\varphi]} - \sum_\ell \mathcal{X}_{j\ell})! \prod_\ell \mathcal{X}_{j\ell}!} (\prod_\ell p_{j\ell}^{\mathcal{X}_{j\ell}})(1 - \sum_\ell p_{j\ell})^{(\mathcal{D}_j^{[\varphi]} - \sum_\ell \mathcal{X}_{j\ell})},$$

where $(\mathcal{D}_j^{[\varphi]} - \sum_\ell \mathcal{X}_{j\ell})$ indicates daily number of non-traveling individuals of compartment $[\varphi]$ staying in subpopulation j. It is noticeable that the population N_j of each subpopulation keeps constant, e.g., $\sum_{[\varphi]} \nabla_j(\mathcal{D}^{[\varphi]}) = 0$, because the passenger flows are balanced on each pair of connections in this article.

Pandemic phases defined by the WHO

1. Interpandemic period

Phase 1: no new influenza virus subtypes circulating among animals have been reported to cause infections in humans.

Phase 2: a new influenza virus subtypes circulating among domesticated or wild animals is known to have caused infection in humans, and is therefore considered a potential pandemic threat.

2. Pandemic alert period

Phase 3: a new influenza virus subtypes has caused sporadic cases or small clusters of disease in people, but has not resulted in human-to-human transmission sufficient to sustain community-level outbreaks.

Phase 4: it is characterized by verified human-to-human transmission of a new influenza virus subtypes able to cause "community-level outbreaks". Though the virus is not well adapted to humans, the ability to cause sustained disease outbreaks in a community marks a significant upwards shift in the risk for a pandemic.

Phase 5: it is characterized by human-to-human spread of the virus into at least two countries in one WHO region. While most countries will not be affected at this stage, the declaration of Phase 5 is a strong signal that a pandemic is imminent.

3. Pandemic period

Phase 6: it is characterized by community level outbreaks in at least one other country in a different WHO region in addition to the criteria defined in Phase 5. This phase indicates that a global pandemic is under way.

Power Law Distribution with an Exponential Cutoff

In fact, little real systems do display a perfect power law pattern for the Zipf's distribution or probability density distribution[1,2]. When an exponential cutoff is added to the power law function, the fit is substantially improved for dozens of systems, e.g., the forest fires, earthquakes, web hits, email networks, sexual contact networks. The cutoff indicates that there is a characteristic scale, and that infrequently super-enormous events are exponentially rare. Strictly speaking, a cutoff power law should always fit the data at least as good as a pure one(just let the cutoff scale go to infinity), thus the power law distribution can be deemed as a subset of the exponentially cutoff power law[2].

The Mass Action Principle

Prof. Hamer postulated that the course of an epidemic depends on the rate of contact between susceptible and infectious individuals. This conception plays a significant role in the study of epidemiology; it is the so-called 'mass action principle' in which the net rate of spread of infection is assumed to be proportional to the product of the density of susceptible persons times the density of infectious individuals[3,4].

1. Newman MEJ (2005) Power laws, Pareto distributions and Zipf's law. Contemporary Physics 46: 323-351.
2. Clauset A, Shalizi CR, and Newman MEJ (2009) Power-law distributions in empirical data. SIAM Review 51, 661-703.
3. Anderson RM and May RM (1991) Infectious Diseases of Humans: Dynamics and Control(Oxford Unvi. Press, Oxford).
4. Hamer WH (1906) The Milroy Lectures On Epidemic disease in England — The evidence of variability and of persistency of type. The Lancet 167: 733-739.
